# N^6^-methyladenosine (m^6^A) RNA modification in gastrointestinal tract cancers: roles, mechanisms, and applications

**DOI:** 10.1186/s12943-019-1099-7

**Published:** 2019-12-07

**Authors:** Bin-bin Hu, Xiao-yan Wang, Xu-Yu Gu, Chen Zou, Zhen-jun Gao, Heng Zhang, Yu Fan

**Affiliations:** 1 Cancer Institue, Affiliated People’s Hospital of Jiangsu University, Zhenjiang, Jiangsu Province, People’s Republic of China; 2 Digestive Department, Suqian Branch Hospital of Jiangsu Province Hospital, Jiangsu Suqian, People’s Republic of China; 3 Digestive Department, Qingpu Branch Hospital of Affiliated Zhongshan Hospital, Fudan University, Shanghai, People’s Republic of China; 4 Department of General Surgery, Nanjing Lishui District People’s Hospital, Zhongda Hospital Lishui Branch, Southeast University, Nanjing, China

## Abstract

Analogous to DNA methylation and histone modification, RNA modification, as another epigenetic layer, plays an important role in many diseases, especially in tumours. As the most common form of RNA modification, m^6^A methylation has attracted increasing research interest in recent years. m^6^A is catalysed by RNA methyltransferases METTL3, METTL14 and WTAP (writers), m^6^A is removed by the demethylases FTO and ALKBH5 (erasers) and interacts with m6A-binding proteins, such as YT521-B homology (YTH) domain-containing proteins. This article reviews recent studies on methylation modification of m^6^A in gastrointestinal tract cancers.

## Introduction

In recent years, epigenetics has attracted the attention of the research community. Epigenetics is a study of reversible, inheritable phenotypes that do not involve changes in nuclear DNA sequences [1]. Although the full scope of epigenetics has not yet been determined, it is generally defined as chemical modification that mainly includes DNA and RNA methylation, histone modification, noncoding RNA modification and chromatin rearrangement. In epigenetic modification, DNA methylation and histone modification have been well studied. For example, 5-methylcytosine methylation in DNA has affected gene expression in many tumours. Significant advances have been achieved in recent years in the study of methylated drugs, such as demethylation drugs Decitabine and Azacitidine and histone deacetylase inhibitor Sedamine, which provides additional strategies for treatment of clinical diseases [2, 3]. In addition to DNA and histone methylation, another level of epigenetic regulation, namely, RNA methylation, has become a hot topic in biosciences over the past decade. Common RNA methylation sites include 5-methylcytosine (m^5^C), 7-methylguanosine (m^7^G), m^1^G, m^2^G, m^6^G, N^1^-methyladenosine (m^1^A) and m^6^A. m^5^C modification promotes splicing and translation [4]. m^1^G, m^2^G and m^1^A modifications at the first or second codon repress protein synthesis [5–7], and tRNA m^7^G methylation is required for mRNA translation into proteins [8]. m^6^A is the most common among various RNA modifications [9] and has critical roles in cancer pathogenesis. In this review, we focus on the relationship between RNA m^6^A methylation and gastrointestinal cancer, especially their role, mechanism and potential clinical application as biomarkers and therapeutic targets for gastrointestinal cancer.

### RNA m^6^A methylation

**Fig. 1:**
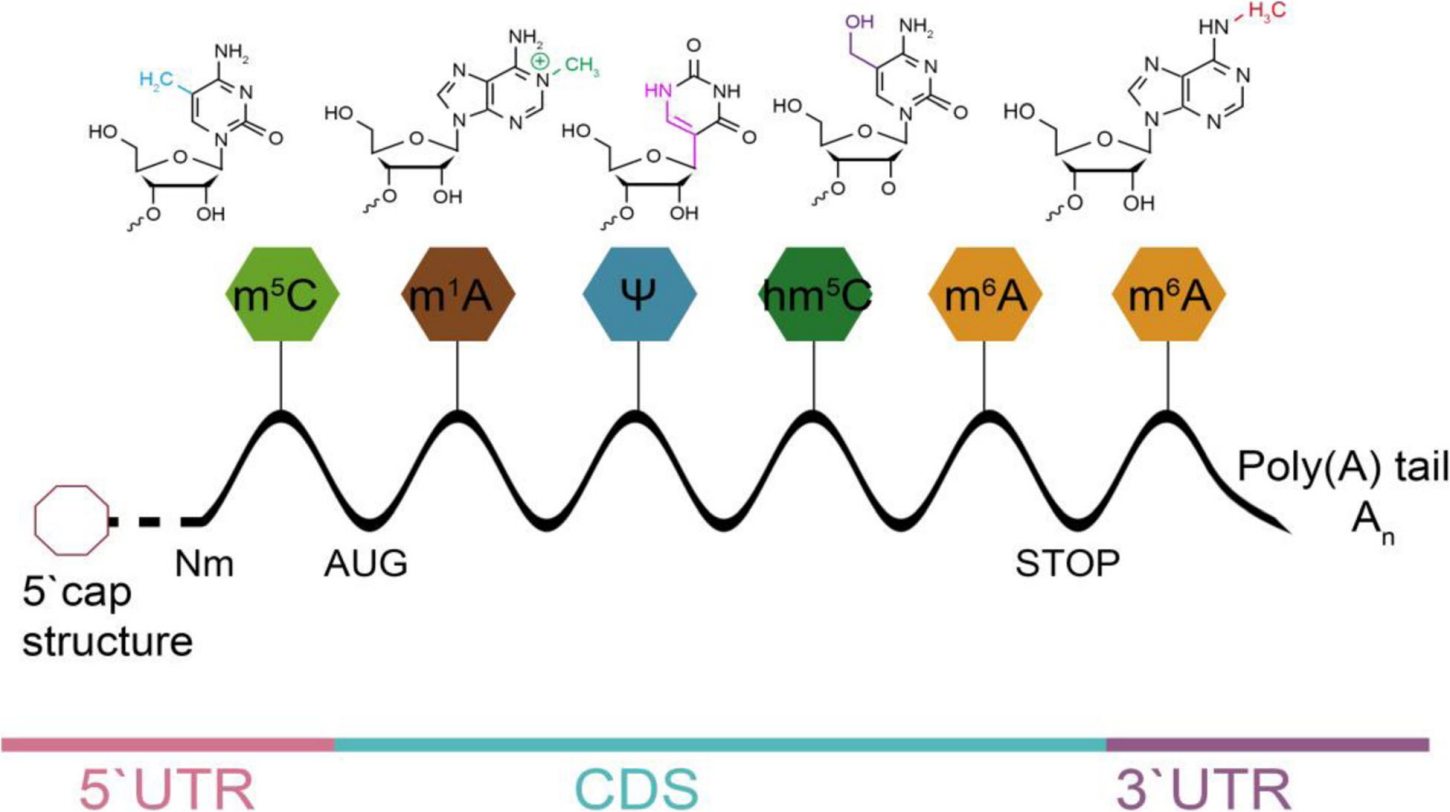
Chemical modification of eukaryotic mRNA. Schematic diagram of common chemical modifications of eukaryotic mRNA transcripts

More than 100 kinds of chemical modifications of RNA, including mRNAs, rRNAs, tRNAs, snRNAs and snoRNAs, have been identified in organisms [10]. Among them, m^6^A, discovered in the 1970s, is the most abundant internal modification of mRNA in most eukaryotes [11] and involves almost all stages of RNA life cycle, including RNA transcription, exporting through nuclear translation and degradation [12–15]. About 0.1–0.4% of adenosine in isolated RNA is modified by m^6^A in mammals [16]. Transcriptome-wide research reveals that m^6^A modification may affect more than 7000 mRNAs in individual transcriptomes of mammalian cells. m^6^A modifications are enriched in the 3′-untranslated regions (UTRs) near the stop codons of mRNAs and with a consensus sequence of RRACH (R = G or A; H = A, C, or U) [17] (Fig. 1). Highly conserved m^6^A is widely present in most eukaryotic species (from yeast, plants and fruit flies to mammals) and viral mRNAs and plays a key regulatory role in post-transcriptional mRNA processing and metabolism. Several lncRNAs also accept m^6^A modification [18].

**Fig. 2:**
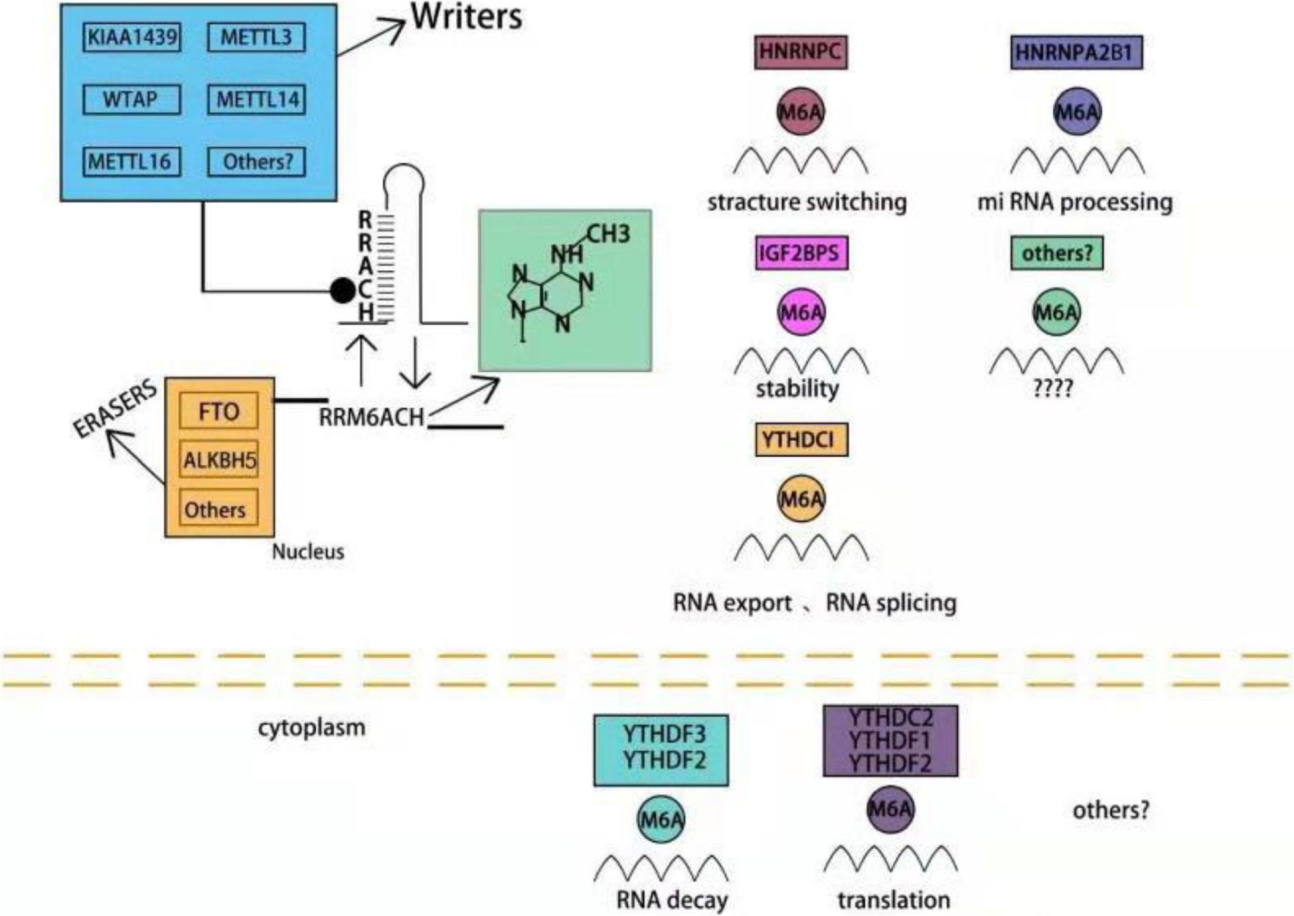
Mechanism of m^6^A methylation m^6^A-RNA methylation is regulated by its “writer”, “eraser” and “reader”, the writer refers to the m^6^A-methylase complex, mainly including METTL3, METTL14, METTL16 and WTAP. The eraser is an m^6^A-demethylase complex, including FTO and ALKBH5. The reader is a protein that binds to m^6^A and includes proteins containing the YTH domain (YTHDF1, YTHDF2, YTHDF3, YTHDC1, YTHDC2), which involving splicing, export, translation, RNA decay

Similar to DNA and histone methylation, m^6^A modifications are dynamic and reversible and exert biological effects that are mainly mediated by ‘writers’, ‘erasers’ and ‘reader’ proteins (Fig. 2). Reversibility means that RNA can be methylated under the action of methyltransferases and demethylated under the action of demethylases; this phenomenon is called dynamic balance. Writers traditionally consist of methyltransferase-like 3 and 14 proteins (METTL3 and METTL14) and their cofactors WTAP (Wilms tumour suppressor-1-associated protein) [19–21]. METTL3 and METTL14 contain an S-adenosylmethionine-binding motif. METTL3 and METTL14 are co-located in nuclear spots and form stable complexes in a 1:1 ratio [22]. METTL3 is a major catalytic enzyme with functions reminiscent of the N^6^-adenine methyltransferase system [23]. METTL14 is a pseudomethyltransferase that stabilizes METTL3 and recognizes target RNA [24]. WTAP is the main regulatory component of the m^6^A methylation complex. WTAP interacts with METTL3 and METTL14 and helps them to be localised in nuclear spots [20]. Writers also include methyltransferase-like 16 (METTL16) [25], KIAA1429 [21] and RBM15 [26]. Demethylation, which is the removal of methyl groups, is also important. Demethylation is achieved by another enzyme family called demethylases (erasers), mainly including FTO and ALKBH5. FTO can sequentially oxidise m^6^A into N^6^-hydroxymethyladeosine and N^6^-formyladenosine, which are moderately stable and can be hydrolysed into adenine. Given that FTO was identified as the first RNA demethylase [27], RNA methylation has gradually gained research attention. ALKBH5, an FTO homologue [28], ensures the equilibrium of m^6^A modification in the transcriptome. In addition to writers and erasers, another important group is readers, which can recognise these modifications, bind to them, and carry out different biological functions [29]. Readers can be recognised by proteins containing the YT521B homology (YTH) domain. The YTH domain in human cells, including YTH domain family (YTHDF1–3), YTH domain-containing 1 (YTHDC1) and YTH domain-containing 2 (YTHDC2), have conserved m^6^A binding domain and preferentially bind to m^6^A-modified RNA in RRm6ACH consensus sequence [11]. Moreover, IGF2BP1–3 is a common reader. YTHDF2, the first characteristic m^6^A reader, can result in the localisation of binding mRNA to the decay site of RNA [30]. YTHDF1 was initially demonstrated to bind to m^6^A sites around the stop codon and improve RNA translation efficiency by interacting with translation initiation factor eif3 [31]. YTHDF3 plays a fine-tuning role in the RNA accessibility of YTHDF1 and YTHDF2.

### Roles of RNA m^6^A in gastrointestinal tract cancers

Emerging evidence suggests that m^6^A is closely associated with the progression of gastrointestinal cancer, including its tumourigenesis, metastasis and angiogenesis. Herein, we briefly review recent studies of m^6^A methylation in gastrointestinal tract cancers (Table 1).

**Table 1:** The roles of RNA m^6^A in gastrointestinal tract cancers

The roles of RNA m^6^A in gastrointestinal tract cancers				
cancer	m^6^A Regulators	Role in cancer	Biological function	Mechanism
Liver cancer	METTL14 [32]	Suppressor gene	Suppresses HCC metastasis	Promotes pri-mia-126 processing
	METTL3 [33]	Oncogene	Promotes HCC growth	Promotes SOCS2 degradation
Gastric cancer	METTL3 [34]	Oncogene	Promotes GC growth	down-regulation of METTL3 leads to inactivation of the AKT signaling pathway
	m^6^A [35]	Suppressor	Suppresses GC cell proliferation and invasiveness	Inhibition of m^6^A activates Wnt and PI3K-AKT signaling
	METTL3 [36]	Oncogene	Promotes GC growth and metastasis	METTL3 knockdown reduced α-smooth muscle actin
colorectal cancer	METTL3 [37]	Oncogene	Promotes CRC growth	prevent SOX2 mRNA degradation
	METTL3 [38]	Suppressor gene	Suppresses CRC proliferation, migration and invasion	activates p38/ERK pathways
	YTHDF1 [39]	Oncogene	–	c-Myc-driven YTHDF1 axis significance
	YTHDF1 [40]	Oncogene	–	YTHDF1 inhibits the activity of Wnt/beta-catenin pathway
pancreatic cancer	YTHDF2 [41]	Oncogene	Promotes the migration and proliferation	YTHDF2 may regulate EMT through YAP signaling.
	FTO [42]	Oncogene	Promotes the migration and proliferation	–

### Liver cancer

Diverse and reversible m^6^A modification on RNAs emerges as a new layer of regulation in liver cancer. In the study of Ma et al. [32], the expression level of m^6^A in HCC decreased firstly, especially in metastatic HCC. METTL14 is an important factor for the aberrant expression of m^6^A. The decrease in METTL14 expression was proven to be related to HCC metastasis in vivo and in vitro. In addition, the target genes of METTL14 during HCC metastasis have been explored. One of these genes is microRNA 126 (micro-126), which plays an important role in cancer metastasis by acting as a tumour inhibitor. The reduction of METTL14 interacts with microprocessor protein DGCR8 and positively regulates pri-microRNA-126 process in an m^6^A-dependent manner, resulting in reduced expression of microRNA-126. Yang and coworkers [33] elucidated that miR-145 can increase the m^6^A level by downregulating YTHDF2 in hepatocellular carcinoma cells. YTHDF2 can recognise mRNA m^6^A site to regulate mRNA degradation [30, 43]. In a previous work [33], miR-145 can inhibit the proliferation of HepG2 cells, suggesting that miR-145-increased methylated mRNAs may be involved in cell apoptosis. Chen and colleagues [44] demonstrated that the expression of METTL3 in human liver cancer is frequently up-regulated and contributes to the progression of liver cancer. METTL3 inhibits the expression of SOCS2 in liver cancer through the m^6^A-YTHDF2-dependent mechanism. Studies have shown that SOCS2 acts as a tumour suppressor but only inhibits the proliferation, migration or drying of different cancer types, including leukaemia, oral squamous cell carcinoma and, more recently HCC [45]. Cheng et al. [46] showed that m^6^A modification also plays an important role in HCC.

### Gastric cancer (GC)

GC is one of the most common malignancies worldwide [47]. Among Chinese men, the morbidity of GC is second. The mortality of GC in the Chinese crowd is also in second [48]. Lin and collaborators [34] found that METTL3, as an oncogene, might be a potential target for treatment of human GC. Zhang and coworkers [35] demonstrated that reduced m^6^A methylation activates oncogenic Wnt/PI3K-AKT signalling and promotes malignant phenotypes in GC cells. Zhang et al. [49] showed that NEAT1 acts as a LncRNA and undergoes m^6^A methylation in GC. As an eraser, ALKBH5 reduced the level of m^6^A methylation of NEAT1, but the expression of ALKBH5 and NEAT1 was positively correlated. Therefore, NEAT1 is upregulated with decreasing m^6^A methylation of NEAT1; this phenomenon promotes the malignant phenotype of GC because NEAT1 can act as a scaffold to affect EZH2 expression, tumour invasion and metastasis. Liu et al. [36] found that METTL3 is a factor of poor prognosis in patients with GC, and the expression level of METTL3 is related to tumour stage and grade. In addition, METTL3 knockdown reduced α-smooth muscle actin. Hence, METTL3 could serve as an oncogene in the tumourigenesis of GC.

### Colorectal cancer (CRC)

CRC is one of the most common malignant tumours in humans and has increasing mortality and morbidity worldwide [50]. Although the level of medical treatment continues to improve, the 5-year relative survival rate of patients is only 64.9% [51]. Li et al. [37] found that METTL3 is highly expressed in metastatic CRC, and its downstream gene is SOX2. SOX2 is recognised as cancer stem-like cells (CSCs) [52]. CSCs are a group of tumour cells with self-renewal ability and multidirectional differentiation potential and related to strong possibility of tumour occurrence and metastasis [53]. Mechanistically, the methylation of SOX2 transcripts in CRC cells, especially in the CD region, leads to the specific recognition of IGF2BP2 by m^6^A readers, thereby preventing the degradation of SOX2 mRNA. In contrast to Li’s conclusion, Deng [38] believed that METTL3 plays an anti-oncogene role in CRC and is a favourable prognostic factor in CRC. Specifically, METTL3 targets downstream p38/ERK signalling pathways. Nishizawa and colleagues [39] elucidated that over-expression of YTHDF1 in CRC cells compared with that in noncancer cells is associated with malignant phenotype and poor prognosis. Moreover, c-myc, an oncogenic transcription factor, activates the transcriptional expression of the YTHDF1 gene. Bai and coworkers [40] found that the mRNA and protein levels of YTHDF1 are overexpressed in CRC. In addition, the expression level of YTHDF1 is related to tumour depth and size. Knockdown of YTHDF1 inhibits the activity of the Wnt/beta-catenin pathway, of which the Wnt pathway component can lead to CRC.

### Pancreatic cancer

Pancreatic neoplasms are one of the few cancers with a mortality rate approaching 100% [54]. The aetiology and screening tests for this highly lethal disease are not well defined; as such, identifying genetic factors that contribute to the development of this cancer is important [55]. Chen and colleagues [41] found that the expression level of YTHDF2 in pancreatic cancer tissues is higher than that in normal tissues at the mRNA and protein levels; moreover, YTHDF2 is an independent influencing factor for the deterioration of patients’ condition. YTHDF2 knockdown promoted the expression of YAP, suggesting that YTHDF2 may regulate EMT through YAP signalling. A large number of studies have shown that EMT can promote the migration and proliferation of tumour cells. Tang [42] demonstrated that FTO, a primary demethylase in vivo, was overexpressed in pancreatic cancer cells compared with that in normal pancreatic epithelial cells. Knockdown of FTO resulted in impaired proliferation and increased apoptosis of pancreatic cancer cells. These observations suggest that FTO is essential for the proliferation of pancreatic cancer cells.

#### Potential application of RNA m^6^A in gastrointestinal tract cancers

##### RNA m^6^A as biomarker in gastrointestinal tract cancers

An increasing number of studies have shown that m^6^A may have great potential as a biomarker for diagnosis, prognosis prediction and therapeutic evaluation of digestive tract tumours. Zhou et al. [56] firstly found that the abnormal expression of METTL3 or YTHDF1 is associated with overall survival in HCC and confirmed its protein expression. Moreover, METTL3 and YTHDF1 were upregulated in HCC. The low METTL3/YTHDF1 group had better prognosis. In summary, the combination of METTL3 and YTHDF1 can be used as biomarker to reflect the malignant degree of liver cancer and evaluate its prognosis. Zhao et al. [57] found that low YTHDF1 expression level is associated with improved survival of patients with HCC. Therefore, YTHDF1 may be a marker of HCC.

In GC, the abnormal expression of FTO has significant prognostic value; hence, FTO may play important roles in GC progression and metastasis [58]. Similarly to the report of Li**,** Xu et al. [59] concluded that FTO expression might play an important role in promoting the occurrence of GC and might be an important molecular marker for the diagnosis and prognosis of this disease. They found that the expression level of FTO is related to poor differentiation, lymph node metastasis, TNM staging and poor prognosis. Therefore, FTO expression may play an important role in promoting the occurrence of GC and may be an important molecular marker for the diagnosis and prognosis of patients with GC. In pancreatic cancer, ALKBH5 is an independent prognostic factor [60].

##### RNA m^6^A as therapeutic targets in in gastrointestinal tract cancers

The important roles of m^6^A in gastrointestinal cancers suggest that it may be exploited as a therapeutic target. For treatment of common digestive tract tumours, radiotherapy and chemotherapy have a pivotal position; however, chemo- and radioresistance lead to unsatisfactory treatment outcomes. Studies have shown that m^6^A methylation is important in treatment of tumours, especially in targeted therapy. In the study of Zhu et al. [61], impaired autophagy degradation of lncRNA ARHGAP5-AS1 in chemoresistant cancer cells may promote chemoresistance. The transcription of ARHGAP5 in the nucleus is activated by recruiting METTL3, and the m^6^A modification of ARHGAP5 is stimulated to stabilise the mRNA of ARHGAP5 in the cytoplasm. Therefore, m^6^A methylation may play an important role in the chemotherapeutic resistance of GC and deserves further study and exploration. Taketo and coworkers [62] established METTL3-knockdown pancreatic cancer cell line by using short hairpin RNA. METTL3-depleted cells showed higher sensitivity to anticancer reagents, such as Gemcitabine, 5-Fluorouracil, Cisplatin and irradiation. In summary, METTL3 is associated with therapeutic resistance and a potential therapeutic target for pancreatic cancer. A previous study suggested that YTHDF1 is a potential therapeutic target for anti-cancer immunotherapy [63]. The down-regulation of the YTHDF1 gene can inhibit tumour proliferation and sensitivity to the exposure of fluorouracil, oxaliplatin and other anticancer drugs [39]. Chen et al. [64] found that rhein, an FTO inhibitor, prevents FTO from binding to m^6^A substrates by binding to the active site of FTO, thereby increasing the cell content of m^6^A in mRNA.

## Discussion

RNA epigenetics has become a hot topic in recent years. Among more than 100 different RNA modifications, m^6^A is the most abundant and influences the processing of lncRNA and miRNA. m^6^A methylation plays an important role in digestive tract tumours. This article describes the role, mechanism and application of m^6^A from the perspective of different tumours in the gastrointestinal tract. However, the current specific mechanism for m^6^A in cancer is unclear because m^6^A methylation has the function of a double-edged sword. High expression levels of m^6^A may lead to the development of certain tumours, but lack of m^6^A modification may lead to the progression of other tumours. (i) For the same tumour, different researchers presented inconsistent results on the expression level of m^6^A because of differences in research samples used. Ma et al. [32] believed that the expression level of m^6^A in liver cancer is reduced, but Yang et al. [33] reported inconsistent results. (ii) m^6^A expression in different tumours has different meanings. (iii) For the same tumour, the same molecule, conclusions may be quite different. For example, for the mechanism of METTL3, Li [37] and Deng [38] reported contradicting results. (iv): For the same tumour, the same molecule, different researchers state contrasting results on its downstream genes and present different mechanisms [37–40]. Previous differences have been noted, but most m^6^A-modified RNAs undergo, rapid journal from RNA processing to degradation and m^6^A controls cell differentiation and pluripotency, which are both associated with cancer progression.

Inflammation is a major cause of liver cancer, GC, CRC and pancreatic cancer. Lipopolysaccharide (LPS) is the main component of the cell wall of Gram-negative bacteria and one of the most effective stimulants of innate immunity. LPS can stimulate macrophages to secrete various inflammatory cytokines, including TNF-α, IL-6 and IL-1β, by activating NF-κB and MAPK pathways, further aggravating the immune response [65]. Yu et al. [66] found the increased expression of YTHDF2 in LPS-stimulated RAW 264.7 cells (RAW 264.7 cells are widely used as a suitable macrophage model for research on inflammation). In addition, inhibition of YTHDF2 enhances the mRNA expression of MAP2K4 and MAP4K4 and triggers the activation of p38, ERK and NF- κB signalling, thereby promoting the expression of TNF-α, IL-1 β, IL-6 and IL-12. These results suggest that YTHDF2 may play a regulatory role in LPS-induced macrophage inflammatory responses. This study provides a potential target for anti-inflammatory therapy and new insights into the mechanisms of inflammation.

At present, most studies on m^6^A methylation report its mechanism and role as a tumour marker. However, few studies have focused on drug therapy. In addition, Future prospects still need to be further explored. Firstly, m^6^A regulates the detailed and precise function of each component in the regulation of cells. Secondly, as a tumour marker, the specificity and sensitivity of m^6^A need further studies. Thirdly, many studies have shown that m^6^A regulatory factors and related pathways can be used as therapeutic targets; however, no specific response has been reported in clinical practice. Future research will demonstrate the feasibility of using m^6^A alone or in combination with other therapies to treat diseases, such as tumours. Fourthly, other components of m^6^A methylation and demethylation and effectors should be identified. For instance, the functions of few m^6^A eraser components (e.g. ALKBH1, ALKBH4, ALKBH6 and ALKBH7) remain unknown and need to be determined.

## Conclusion

Given that m^6^A methylation plays an important role in a variety of digestive tract tumors, m^6^A modification can serve as a diagnostic/prognostic target. Due to various related factors, the results of many researchers are sometimes contradictory. This requires more multi-center and large-scale research to further explore, so as to lay a foundation for accurate treatment of human tumors.

## Data Availability

Not applicable.

## Abbreviations

ALKBH5: A-ketoglutarate-dependent dioxygenase alkB homolog 5
CRC: Colorectal cancer
DGCR8: DiGeorge Syndrome Crisis Area Gene 8
EMT: Epithelial-Mesenchymal Transition
FTO: Obesity-associated protein
HCC: Hepatic cell carcinoma
m^6^A: N^6^-methyladenosine
METTL14: Methyltransferase-like 14
METTL3: Methyltransferase-like 3
SOCS2: Suppressor Of Cytokine Signalling 2
WTAP: Wilms tumor suppressor-1-associated protein
YTHDC1: YTH domain-containing 1
YTHDC2: YTH domain-containing 2
YTHDF1: YTH N^6^-methyladenosine RNA binding protein 1
YTHDF2: YTH N6-methyladenosine RNA binding protein 2
YTHDF3: YTH N6-methyladenosine RNA binding protein 3
